# Differential induction of CCL5 by pathogenic and non-pathogenic strains of West Nile virus in brain endothelial cells and astrocytes

**DOI:** 10.1099/vir.0.060558-0

**Published:** 2014-04

**Authors:** Katherine L. Hussmann, Brenda L. Fredericksen

**Affiliations:** 1 Maryland Pathogen Research Institute, University of Maryland, College Park, MD 20742, USA; 2 Department of Cell Biology and Molecular Genetics, University of Maryland, College Park, MD 20742, USA

## Abstract

The neuroinflammatory response to West Nile virus (WNV) infection can be either protective or pathological depending on the context. Although several studies have examined chemokine profiles within brains of WNV-infected mice, little is known about how various cell types within the central nervous system (CNS) contribute to chemokine expression. Here, we assessed chemokine expression in brain microvascular endothelial cells and astrocytes, which comprise the major components of the blood–brain barrier (BBB), in response to a non-pathogenic (WNV-MAD78) and a highly pathogenic (WNV-NY) strain of WNV. Higher levels of the chemokine CCL5 were detected in WNV-MAD78-infected brain endothelial monolayers compared with WNV-NY-infected cells. However, the opposite profile was observed in WNV-infected astrocytes, indicating that pathogenic and non-pathogenic strains of WNV provoke different CCL5 profiles at the BBB. Thus, cells comprising the BBB may contribute to a dynamic pro-inflammatory response within the CNS that evolves as WNV infection progresses.

West Nile virus (WNV) is a positive-strand RNA virus in the family *Flaviviridae*. Prior to the 1990s, WNV infections were typically asymptomatic or associated with a self-limiting mild febrile illness known as West Nile fever; however, recent outbreaks of WNV infection in the Western hemisphere and Europe have been associated with an increased incidence of neuroinvasive disease, including meningitis, encephalitis and acute flaccid paralysis (http://www.cdc.gov/ncidod/dvbid/westnile/index.htm) (Papa *et al.*, 2010, 2011).

One of the hallmarks of WNV neuroinvasion is the recruitment of inflammatory leukocytes into the central nervous system (CNS) (Omalu *et al.*, 2003). Leukocyte infiltration is critical for clearance of WNV from the CNS and is a major determinant for survival. The process of leukocyte infiltration into infected tissues is a highly regulated, multistep process mediated by a family of chemoattractant cytokines known as chemokines (Carman, 2009; Middleton *et al.*, 2002). Chemokines function by rapidly attracting a subset of leukocytes that express the appropriate cognate receptor. Therefore, chemokine expression patterns determine the type and abundance of cells attracted to the site of infection. Several studies have demonstrated that the chemokine receptors CCR2 and CCR5 are essential for leukocyte accumulation within the brains of WNV-infected animals and for survival (Glass *et al.*, 2005; Lim *et al.*, 2011). Although the cognate chemokines CCL2 and CCL5 have been detected in the brains of WNV-infected mice (Glass *et al.*, 2005; Klein *et al.*, 2005; Shirato *et al.*, 2004), the specific cell types that express these chemokines have not been characterized. Here, we examined the CCL2 and CCL5 expression profiles in brain microvascular endothelial cells and astrocytes, which together comprise the blood–brain barrier (BBB), in response to WNV infection.

Human brain microvascular endothelial cells (HBMECs) (Hussmann *et al.*, 2013; Stins *et al.*, 2001) were infected at an m.o.i. of 0.1 with either a non-pathogenic lineage 2 African isolate, WNV-MAD78 (Beasley *et al.*, 2002), or a highly pathogenic lineage 1 North American strain, WNV-NY (Shi *et al.*, 2002). Total cellular RNA and culture supernatants were collected at the indicated times and analysed by quantitative reverse transcription-PCR and ELISA, respectively (Fig. 1a–d). Consistent with a previous report (Chui & Dorovini-Zis, 2010), CCL2 was expressed constitutively in mock-infected HBMECs (Fig. 1a, c). Moreover, WNV infection did not significantly alter the level of CCL2 mRNA or secreted protein in HBMECs. As stimulation of endothelial cells was shown previously to induce the redistribution of constitutively expressed CCL2 to the cell surface (Chui & Dorovini-Zis, 2010), we also assessed the CCL2 expression pattern throughout the endothelial cell layer during WNV infection using confocal microscopy. HBMECs were grown on fibronectin-coated Transwell supports (BD Biosciences; 3 µm pores) and mock-infected or infected with WNV at an m.o.i. of 0.1. At 48 h post-inoculation, the Transwell supports were fixed with paraformaldehyde, permeabilized with Triton X-100 to allow the detection of cytokines within secretory vesicles and probed for CCL2. The intensity of CCL2 staining within individual *z*-sections was determined using the image processing program ImageJ (Schneider *et al.*, 2012). Unlike other stimuli (Chui & Dorovini-Zis, 2010), WNV infection did not induce CCL2 redistribution within the cells (data not shown) or increase the overall intensity of CCL2 staining compared with mock-infected cells (Fig. 1e). Thus, WNV infection does not alter the CCL2 expression pattern within HBMECs.

**Fig. 1.**
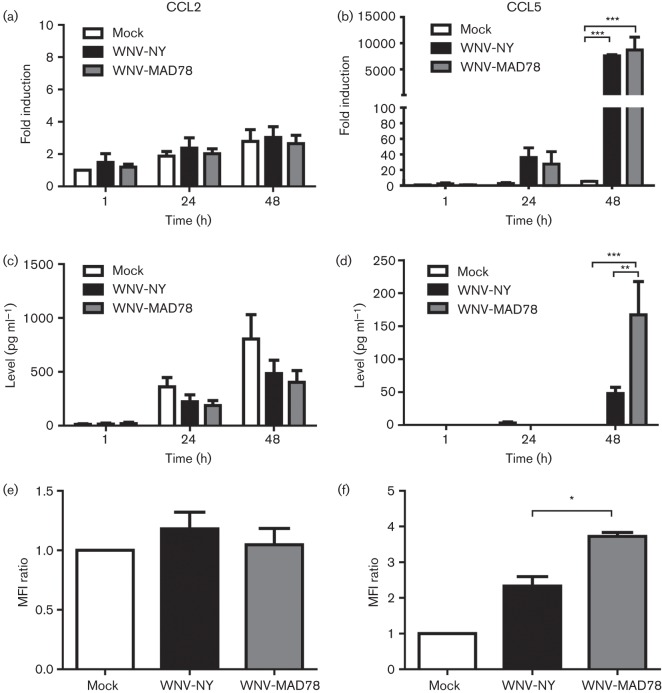
WNV-induced chemokine expression in HBMECs. (a, b) Expression of CCL2 and CCL5 mRNA. CCL2 (a) and CCL5 (b) mRNA levels were determined by quantitative reverse transcription-PCR using primers specific for CCL2 (forward, 5′-TCTCTGCCGCCCTTCTGTG-3′; reverse, 5′-GCTTCTTTGGGACACTTGCTGCTG-3′) and CCL5 (forward, 5′-CTCTGTGACCAGGAAGGAAGT-3′; reverse, 5′-GGGTTGAGACGGCGGAAG-3′). Chemokine levels were normalized to glyceraldehyde 3-phosphate dehydrogenase gene expression and the fold induction compared with mock-infected HBMECs at 1 h post-infection was determined. Values represent the mean±se from at least three separate experiments. (c, d) Secreted chemokine levels. Levels of CCL2 (c) and CCL5 (d) in culture supernatants were determined by ELISA (BD Biosciences and R&D Systems, respectively). Values represent the mean±se from at least three independent experiments. (e, f) Confocal analysis of chemokine expression. CCL2 (e) was detected with polyclonal antisera (Abcam) and DyLight 488-conjugated anti-rabbit secondary antibody (Jackson Immunolabs). CCL5 (f) was detected with polyclonal antisera (R&D Systems) followed by a Alexa Fluor 633-conjugated anti-goat secondary antibody (Invitrogen). Images were captured on a Zeiss LSM710 confocal microscope at ×40 magnification. The ratio of the total mean fluorescence intensity (MFI) of all *z*-sections of WNV-infected cells compared with mock-infected HBMECs is presented. Data represent the mean±se of at least two independent experiments. One-way ANOVA was performed to determine significance: *P<0.05, **P<0.01, ***P<0.005.

Unlike CCL2, CCL5 mRNA expression was upregulated ~6000-fold in both WNV-NY- and WNV-MAD78-infected HBMECs compared with mock-infected HBMECs (Fig. 1b, *P*<0.005). However, the level of secreted and cell-associated CCL5 was significantly higher in WNV-MAD78-infected cultures compared with WNV-NY-infected cultures [Fig. 1d (3.5-fold, *P*<0.01) and f (1.6-fold, *P*<0.05)], suggesting that CCL5 expression is regulated at both the transcriptional and translational level within these cells. Moreover, these findings suggest that pathogenic and non-pathogenic strains of WNV differentially induce CCL5 expression in HBMECs.

Astrocytes, the second major cellular component of the BBB, play a central role in mediating neuroinflammation in response to many viruses (Cheeran *et al.*, 2005; Eugenin *et al.*, 2011; Louboutin & Strayer, 2012; Pozner *et al.*, 2008; van Marle *et al.*, 2007). Therefore, we also examined CCL2 and CCL5 expression in primary human brain cortical astrocytes (HBCAs) (Cell Systems) during WNV infection (m.o.i. 0.01). As observed in HBMECs, CCL2 was expressed constitutively in HBCAs and WNV infection did not alter the level of secreted CCL2 (Fig. 2a). However, in contrast to HBMECs, HBCAs secreted significantly more CCL5 in response to WNV-NY infection compared with WNV-MAD78 infection (Fig. 2b, *P*<0.005). Together, these findings suggest that the chemokine profiles induced by pathogenic and non-pathogenic strains of WNV vary as the infection progresses within the BBB from endothelial cells into astrocytes. The higher levels of CCL5 detected in WNV-NY-infected HBCA cultures were probably due to the enhanced ability of WNV-NY to spread from cell to cell within the HBCA monolayer compared with WNV-MAD78 (Hussmann *et al.*, 2013). Indeed, when CCL5 levels were normalized to the number of infected cells as determined by flow cytometry, we observed that WNV-MAD78- and WNV-NY-infected HBCAs secreted similar levels per cell (~0.547 and ~0.146 pg per cell, respectively). Notably, the overall expression of CCL5 in HBCAs in response to WNV-NY and WNV-MAD78 infection (around ten- and twofold, respectively) was greater than that induced in HBMECs (compare Fig. 1d with Fig. 2b). This is despite the fact that HBCAs are less permissive for WNV replication than HBMECs (Hussmann *et al.*, 2013).

**Fig. 2.**
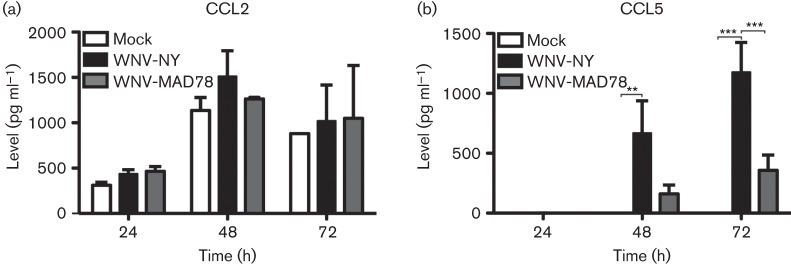
CCL2 and CCL5 expression in WNV-infected HBCAs. The levels of secreted CCL2 (a) or CCL5 (b) were determined by ELISA (BD Biosciences and R&D Systems, respectively). Values represent the mean±se from at least three independent experiments. One-way ANOVA was performed to determine significance: **P<0.01, ***P<0.005.

The primary function of chemokines is to regulate leukocyte migration. Therefore, we assessed whether WNV-induced CCL5 expression enhanced the ability of THP-1 cells, a CCR5-expressing monocyte cell line (data not shown and Swan *et al.*, 2006), to adhere to and traverse an intact HBMEC monolayer. THP-1 cells labelled with CellTracker Green CMFDA (2 µM; Invitrogen) were incubated with TNF-α-treated (100 ng), mock-infected or WNV-infected HBMECs for 30 min at 37 °C, 5 % CO_2_. The monolayers were washed vigorously to remove unbound cells prior to being fixed with paraformaldehyde and the attached THP-1 cells were visualized by microscopy. THP-1 adherence was similar in mock- and WNV-NY-infected cultures (Fig. 3a). However, monocyte attachment was increased twofold in WNV-MAD78-infected cultures, which is consistent with the observation that this virus induced higher levels of CCL5 expression in HBMECs compared with WNV-NY. As expected, high levels of THP-1 cells bound to control cells treated with TNF-α (Liu & Dorovini-Zis, 2012). Together, these findings suggest that the chemokine response in HBMEC monolayers to non-pathogenic strains of WNV may be more efficient at attracting leukocytes compared with highly pathogenic strains of WNV.

**Fig. 3.**
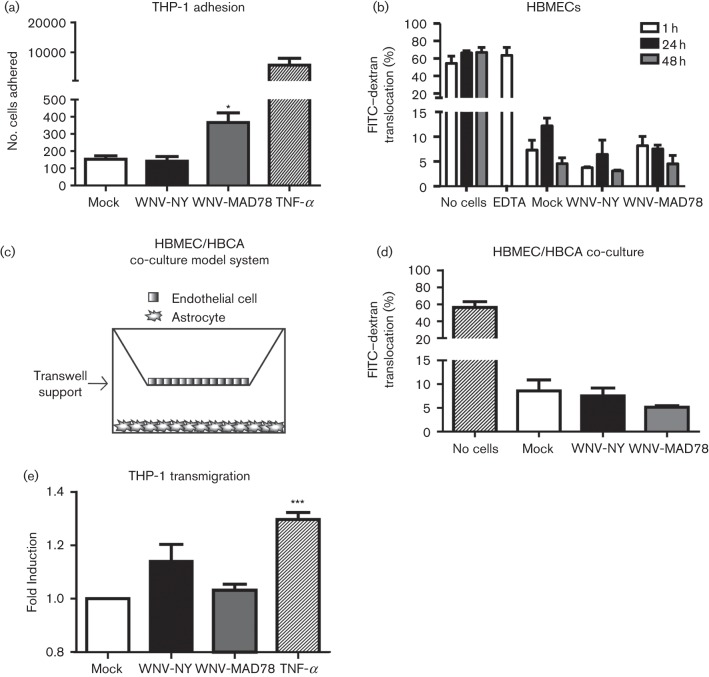
Effect of WNV replication on THP-1 adhesion and transmigration. (a) Adherence of THP-1 cells to HBMECs. Results represent the mean±se of three independent experiments. (b, d) FITC-dextran translocation across HBMEC monolayers in the absence (b) or presence (d) of HBCAs. The level of fluorescence in abluminal supernatants was determined in triplicate. Values represent the mean±se from at least two independent experiments. (c) Schematic of HBMEC/HBCA co-culture assembly in Transwells. (e) Transmigration of THP-1 cells through the HBMEC layer of HBMEC/HBCA co-cultures. Total fluorescence present in abluminal lysates was determined in triplicate. Values were normalized to the total fluorescence present in the mock wells. Data represent the mean±se of at least three independent experiments. One-way ANOVA was performed to determine statistical significance: *P<0.05, ***P<0.005.

In order for circulating leukocytes to facilitate viral clearance within the CNS, they must also traverse the endothelial layer of the BBB. In addition to attracting leukocytes to the site of infection, chemokines can also facilitate infiltration into the CNS by promoting the disruption of endothelial cell–cell junctions (Roberts *et al.*, 2012). As WNV infection induced CCL5 expression in HBMECs, we assessed the integrity of the WNV-infected HBMEC monolayer by quantifying the level of FITC-labelled dextran translocation across a Transwell support (Fig. 3b). FITC–dextran (100 µM, 4 kDa; Sigma) was added to the upper (luminal) chamber at the indicated times post-infection and the amount of FITC–dextran present in the lower (abluminal) chamber supernatants after a 1 h incubation at 37 °C, 5 % CO_2_ was determined using a FLUOstar Omega fluorescence microtitre plate reader (BMG Labtech). Approximately 70 % of the FITC–dextran was detected in the lower chamber of control wells lacking cells or containing HBMEC monolayers treated with EDTA to disrupt tight junctions. In contrast, only 5–10 % of the FITC–dextran was detected in the lower chamber of mock-, WNV-NY- and WNV-MAD78-infected cultures, confirming that WNV infection of the brain endothelial cells is not sufficient to induce disruption of the monolayer (Verma *et al.*, 2009).

In many cases, viral disruption of the BBB is the result of breakdown of the endothelial tight junctions by matrix metalloproteinases secreted from infected astrocytes (Roe *et al.*, 2012; Spindler & Hsu, 2012; Verma *et al.*, 2010). We therefore assessed the effect of WNV replication on the integrity of a simplified *in vitro* model of the BBB consisting of HBMECs grown on 8 µm Transwell inserts and HBCAs on the bottom well (Fig. 3c). HBMECs were infected with WNV at an m.o.i. of 0.1 and incubated for 72 h, which we have demonstrated previously was sufficient time for the infection to progress to the HBCA monolayer (Hussmann *et al.*, 2013). The inclusion of astrocytes in the Transwell system did not enhance FITC–dextran translocation across the HBMEC monolayer, indicating that the monolayer remained intact (Fig. 3d). Thus, the initial infiltration of leukocytes into the CNS is probably due to the active recruitment by chemokines rather than passive migration resulting from the loss of integrity of the BBB. To determine whether chemokines expressed by brain endothelial cells and astrocytes in response to WNV infection facilitate leukocyte traversal of the endothelial monolayer, we assessed the effect of WNV replication on THP-1 transmigration across an HBMEC monolayer. Co-cultures of HBMECs and HBCAs were assembled in Transwells as described previously in Figure 3c and infected at an m.o.i. of 0.1 with either WNV-NY or WNV-MAD78. At 72 h post-inoculation, fluorescently labelled THP-1 monocytes were added to the luminal chamber and cultures were incubated for 6 h at 37 °C, 5 % CO_2_ to allow transmigration. Abluminal lysates were prepared by adding Triton X-100 (2 %) to abluminal supernatants and total fluorescence determined using a FLUOstar Omega fluorescence microtitre plate reader (Fig. 3e). Whilst increased levels of fluorescence were detected in control cells treated with TNF-α (250 ng ml^−1^), WNV infection did not significantly enhance THP-1 migration compared with mock treated co-cultures. This suggests that the chemokine profiles induced in response to WNV infection of HBMECs and HBCAs were not sufficient to mediate monocyte transmigration across the endothelial cell monolayer.

In conclusion, our data suggest that pathogenic and non-pathogenic strains of WNV differentially induce the pro-inflammatory chemokine CCL5 at the BBB, although neither virus substantially induced CCL2. During initial infection of brain endothelial cells, the enhanced chemokine expression in response to WNV-MAD78 may stimulate the rapid recruitment of circulating leukocytes to the BBB. However, the induction of CCL5 in response to WNV infection was not sufficient to promote leukocyte transmigration across the endothelial layer in an *in vitro* model of the BBB containing both endothelial cells and astrocytes. Other cell types within the CNS, such as neurons or activated microglia, may therefore be responsible for the establishment of the chemokine gradients that attract immune cells into the CNS during WNV infection. Likewise, WNV replication did not affect the integrity of the HBMEC monolayer, suggesting that additional host factors are required for the breakdown of the BBB that occurs *in vivo* late in infection (Roe *et al.*, 2012; Wang *et al.*, 2004, 2008). Nonetheless, chemokine expression by endothelial cells and astrocytes early during WNV infection may still play an important role in the neuroimmune response. Increased local expression of CCL5 may function to enhance recruitment and activation of resident microglial cells to the site of infection, thereby promoting the clearance of free virus as well as infected cells in the CNS through an inside-out mechanism or by mediating antigen presentation to infiltrating lymphocytes. There is also increasing evidence that chemokines are multifunctional proteins with activities beyond the recruitment and activation of immune cells. Indeed, CCL5 has been reported to have both neuroprotective (Bruno *et al.*, 2000) and direct antiviral activities (Nakayama *et al.*, 2006). Further analysis describing how early activation of the neuroimmune response may contribute to neuroprotection or pathogenicity is necessary to determine the function of CCL5 produced by the BBB during WNV infection.

## References

[r1] Beasley D. W. Li L. Suderman M. T. Barrett A. D. **(**2002**).** Mouse neuroinvasive phenotype of West Nile virus strains varies depending upon virus genotype. Virology 296, 17–23 10.1006/viro.2002.1372 12036314

[r2] Bruno V. Copani A. Besong G. Scoto G. Nicoletti F. **(**2000**).** Neuroprotective activity of chemokines against *N*-methyl-d-aspartate or β-amyloid-induced toxicity in culture. Eur J Pharmacol 399, 117–121 10.1016/S0014-2999(00)00367-8 10884510

[r3] Carman C. V. **(**2009**).** Mechanisms for transcellular diapedesis: probing and pathfinding by ‘invadosome-like protrusions’. J Cell Sci 122, 3025–3035 10.1242/jcs.047522 19692589

[r4] Cheeran M. C. Hu S. Sheng W. S. Rashid A. Peterson P. K. Lokensgard J. R. **(**2005**).** Differential responses of human brain cells to West Nile virus infection. J Neurovirol 11, 512–524 10.1080/13550280500384982 16338745

[r5] Chui R. Dorovini-Zis K. **(**2010**).** Regulation of CCL2 and CCL3 expression in human brain endothelial cells by cytokines and lipopolysaccharide. J Neuroinflammation 7, 1 10.1186/1742-2094-7-1 20047691 PMC2819252

[r6] Eugenin E. A. Clements J. E. Zink M. C. Berman J. W. **(**2011**).** Human immunodeficiency virus infection of human astrocytes disrupts blood–brain barrier integrity by a gap junction-dependent mechanism. J Neurosci 31, 9456–9465 10.1523/JNEUROSCI.1460-11.2011 21715610 PMC3132881

[r7] Glass W. G. Lim J. K. Cholera R. Pletnev A. G. Gao J. L. Murphy P. M. **(**2005**).** Chemokine receptor CCR5 promotes leukocyte trafficking to the brain and survival in West Nile virus infection. J Exp Med 202, 1087–1098 10.1084/jem.20042530 16230476 PMC2213214

[r8] Hussmann K. L. Samuel M. A. Kim K. S. Diamond M. S. Fredericksen B. L. **(**2013**).** Differential replication of pathogenic and nonpathogenic strains of West Nile virus within astrocytes. J Virol 87, 2814–2822 10.1128/JVI.02577-12 23269784 PMC3571364

[r9] Klein R. S. Lin E. Zhang B. Luster A. D. Tollett J. Samuel M. A. Engle M. Diamond M. S. **(**2005**).** Neuronal CXCL10 directs CD8^+^ T-cell recruitment and control of West Nile virus encephalitis. J Virol 79, 11457–11466 10.1128/JVI.79.17.11457-11466.2005 16103196 PMC1193600

[r10] Lim J. K. Obara C. J. Rivollier A. Pletnev A. G. Kelsall B. L. Murphy P. M. **(**2011**).** Chemokine receptor Ccr2 is critical for monocyte accumulation and survival in West Nile virus encephalitis. J Immunol 186, 471–478 10.4049/jimmunol.1003003 21131425 PMC3402345

[r11] Liu K. K. Dorovini-Zis K. **(**2012**).** Differential regulation of CD4^+^ T cell adhesion to cerebral microvascular endothelium by the β-chemokines CCL2 and CCL3. Int J Mol Sci 13, 16119–16140 10.3390/ijms131216119 23203188 PMC3546682

[r12] Louboutin J. P. Strayer D. S. **(**2012**).** Blood–brain barrier abnormalities caused by HIV-1 gp120: mechanistic and therapeutic implications. ScientificWorldJournal 2012, 482575 10.1100/2012/482575 22448134 PMC3289936

[r13] Middleton J. Patterson A. M. Gardner L. Schmutz C. Ashton B. A. **(**2002**).** Leukocyte extravasation: chemokine transport and presentation by the endothelium. Blood 100, 3853–3860 10.1182/blood.V100.12.3853 12433694

[r14] Nakayama T. Shirane J. Hieshima K. Shibano M. Watanabe M. Jin Z. Nagakubo D. Saito T. Shimomura Y. Yoshie O. **(**2006**).** Novel antiviral activity of chemokines. Virology 350, 484–492 10.1016/j.virol.2006.03.004 16603217

[r15] Omalu B. I. Shakir A. A. Wang G. Lipkin W. I. Wiley C. A. **(**2003**).** Fatal fulminant pan-meningo-polioencephalitis due to West Nile virus. Brain Pathol 13, 465–472 10.1111/j.1750-3639.2003.tb00477.x 14655752 PMC8095851

[r16] Papa A. Danis K. Baka A. Bakas A. Dougas G. Lytras T. Theocharopoulos G. Chrysagis D. Vassiliadou E. **& other authors (**2010**).** Ongoing outbreak of West Nile virus infections in humans in Greece, July-August 2010. Euro Surveill 15 20807489 10.2807/ese.15.34.19644-en

[r17] Papa A. Bakonyi T. Xanthopoulou K. Vázquez A. Tenorio A. Nowotny N. **(**2011**).** Genetic characterization of West Nile virus lineage 2, Greece, 2010. Emerg Infect Dis 17, 920–922 10.3201/eid1705.101759 21529413 PMC3321789

[r18] Pozner R. G. Collado S. Jaquenod de Giusti C. Ure A. E. Biedma M. E. Romanowski V. Schattner M. Gómez R. M. **(**2008**).** Astrocyte response to Junín virus infection. Neurosci Lett 445, 31–35 10.1016/j.neulet.2008.08.059 18771707

[r19] Roberts T. K. Eugenin E. A. Lopez L. Romero I. A. Weksler B. B. Couraud P. O. Berman J. W. **(**2012**).** CCL2 disrupts the adherens junction: implications for neuroinflammation. Lab Invest 92, 1213–1233 10.1038/labinvest.2012.80 22641100 PMC3409314

[r20] Roe K. Kumar M. Lum S. Orillo B. Nerurkar V. R. Verma S. **(**2012**).** West Nile virus-induced disruption of the blood–brain barrier in mice is characterized by the degradation of the junctional complex proteins and increase in multiple matrix metalloproteinases. J Gen Virol 93, 1193–1203 10.1099/vir.0.040899-0 22398316 PMC3755517

[r21] Schneider C. A. Rasband W. S. Eliceiri K. W. **(**2012**).** NIH Image to ImageJ: 25 years of image analysis. Nat Methods 9, 671–675 10.1038/nmeth.2089 22930834 PMC5554542

[r22] Shi P. Y. Tilgner M. Lo M. K. Kent K. A. Bernard K. A. **(**2002**).** Infectious cDNA clone of the epidemic West Nile virus from New York City. J Virol 76, 5847–5856 10.1128/JVI.76.12.5847-5856.2002 12021317 PMC136194

[r22b] Shirato K. Kimura T. Mizutani T. Kariwa H. Takashima J. **(**2004**).** Different chemokine expression in lethal and non-lethal murine West Nile virus infection. J Med Virol 74, 507–513 10.1128/JVI.76.12.5847-5856.2002 15368509

[r23] Spindler K. R. Hsu T. H. **(**2012**).** Viral disruption of the blood–brain barrier. Trends Microbiol 20, 282–290 10.1016/j.tim.2012.03.009 22564250 PMC3367119

[r24] Stins M. F. Badger J. Sik Kim K. **(**2001**).** Bacterial invasion and transcytosis in transfected human brain microvascular endothelial cells. Microb Pathog 30, 19–28 10.1006/mpat.2000.0406 11162182

[r25] Swan C. H. Bühler B. Steinberger P. Tschan M. P. Barbas C. F. III Torbett B. E. **(**2006**).** T-cell protection and enrichment through lentiviral CCR5 intrabody gene delivery. Gene Ther 13, 1480–1492 10.1038/sj.gt.3302801 16738691

[r26] van Marle G. Antony J. Ostermann H. Dunham C. Hunt T. Halliday W. Maingat F. Urbanowski M. D. Hobman T. **& other authors (**2007**).** West Nile virus-induced neuroinflammation: glial infection and capsid protein-mediated neurovirulence. J Virol 81, 10933–10949 10.1128/JVI.02422-06 17670819 PMC2045515

[r27] Verma S. Lo Y. Chapagain M. Lum S. Kumar M. Gurjav U. Luo H. Nakatsuka A. Nerurkar V. R. **(**2009**).** West Nile virus infection modulates human brain microvascular endothelial cells tight junction proteins and cell adhesion molecules: transmigration across the *in vitro* blood–brain barrier. Virology 385, 425–433 10.1016/j.virol.2008.11.047 19135695 PMC2684466

[r28] Verma S. Kumar M. Gurjav U. Lum S. Nerurkar V. R. **(**2010**).** Reversal of West Nile virus-induced blood–brain barrier disruption and tight junction proteins degradation by matrix metalloproteinases inhibitor. Virology 397, 130–138 10.1016/j.virol.2009.10.036 19922973 PMC3102050

[r29] Wang T. Town T. Alexopoulou L. Anderson J. F. Fikrig E. Flavell R. A. **(**2004**).** Toll-like receptor 3 mediates West Nile virus entry into the brain causing lethal encephalitis. Nat Med 10, 1366–1373 10.1038/nm1140 15558055

[r30] Wang P. Dai J. Bai F. Kong K. F. Wong S. J. Montgomery R. R. Madri J. A. Fikrig E. **(**2008**).** Matrix metalloproteinase 9 facilitates West Nile virus entry into the brain. J Virol 82, 8978–8985 10.1128/JVI.00314-08 18632868 PMC2546894

